# Myelin Oligodendrocyte Glycoprotein Antibody-Associated Disease Onset in Pregnancy: A Case Report

**DOI:** 10.7759/cureus.108214

**Published:** 2026-05-04

**Authors:** Adrián Infante Valenzuela, Hugo A Saldaña Velázquez, Adlen S Martínez Torres, Diego A García Garza, Irma A Villarreal Ondarza, Fernanda Flores Alfaro, Sergio Saldivar Dávila, Beatriz E Chávez Luévanos, Fernando Góngora-Rivera

**Affiliations:** 1 Neurology, Autonomous University of Nuevo León, Monterrey, MEX

**Keywords:** methylprednisolone, mogad, optic neuritis, plasma exchange, pregnancy

## Abstract

Myelin oligodendrocyte glycoprotein antibody-associated disease (MOGAD) is an autoimmune demyelinating disorder of the central nervous system in which optic neuritis is a frequent manifestation. Its presentation during pregnancy may pose a diagnostic challenge because bilateral optic disc edema and headache can mimic idiopathic intracranial hypertension or hypertensive disorders of pregnancy.

We report a 39-year-old primigravid woman at 31 weeks of gestation with gestational diabetes and class III obesity who presented with progressive bifrontal headache followed by transient bilateral blurred vision, dyschromatopsia, and sudden severe visual loss in the right eye. Ophthalmologic examination showed bilateral papilledema, a relative afferent pupillary defect in the right eye, and reduced visual acuity. Ultrasound demonstrated an enlarged optic nerve sheath diameter, while brain and spine magnetic resonance imaging showed no major abnormalities. Visual evoked potentials revealed bilateral conduction delay, more severe in the right eye. Lumbar puncture showed a normal opening pressure (19.5 cmH2O) and normal cerebrospinal fluid cytochemistry, but cerebrospinal fluid-restricted oligoclonal bands were detected. Given the clinical suspicion of inflammatory optic neuropathy, serum testing for demyelinating disorders was performed and was positive for anti-myelin oligodendrocyte glycoprotein (MOG) antibodies, whereas aquaporin-4 and systemic autoimmune antibodies were negative. The patient was treated with intravenous methylprednisolone followed by therapeutic plasma exchange because of limited initial response, with progressive visual recovery and no maternal or fetal complications.

This case highlights that MOG-associated optic neuritis should be considered in pregnant patients with acute visual loss and bilateral optic disc edema. Intracranial hypertension was ruled out, and paraclinical studies concluded bilateral inflammatory optic neuritis. Early recognition, serologic confirmation, and timely immunotherapy are essential to improve visual outcomes. Careful postpartum follow-up is warranted because relapse risk may increase after delivery.

## Introduction

Myelin oligodendrocyte glycoprotein antibody-associated disease (MOGAD) is an inflammatory demyelinating disorder of the central nervous system (autoimmune condition targeting the myelin that covers structures of the nervous system) characterized by antibodies directed against myelin oligodendrocyte glycoprotein (MOG), as described in previous studies [1,2]. Since the identification of MOG-immunoglobulin G (MOG-IgG) as a distinctive biomarker, MOGAD has been recognized as a separate clinical entity within the spectrum of autoimmune demyelinating diseases. In adults, optic neuritis represents one of the most common clinical manifestations of the disease, as reported in clinical series and cohort studies [3,4].

Optic neuritis associated with MOG antibodies (MOG-ON) often presents with clinical and radiological features that differ from those observed in multiple sclerosis and aquaporin-4 (channels located primarily in the podocytes of astrocytes) antibody-associated neuromyelitis optic spectrum disorder (AQP4-ON) [5]. Typical findings include bilateral optic nerve involvement, marked optic disc edema, and prominent optic nerve lesions on orbital magnetic resonance imaging (MRI), as described in the literature [6]. Although visual recovery is frequently favorable after corticosteroid treatment, relapses may occur and can lead to cumulative optic nerve injury [7].

The evaluation of acute visual loss during pregnancy represents a particular diagnostic challenge, as several neurological and ophthalmological conditions must be considered. In pregnant patients presenting with bilateral optic disc edema, the differential diagnosis often includes idiopathic intracranial hypertension (IIH) and hypertensive disorders of pregnancy. IIH is more common in pregnancy, either pre-existing and exacerbated by weight gain and hormonal changes during this period or arising de novo. In addition, inflammatory optic neuropathies such as MOG-ON may present with overlapping clinical features, which can further complicate the diagnostic process.

Evidence regarding the clinical course of MOGAD during pregnancy remains limited. While some studies suggest that disease activity may decrease during gestation, the postpartum period could be associated with an increased risk of relapse [8]. Hormonal changes occurring during pregnancy may modify the course of autoimmune diseases. The pregnancy-related relapses were characterized by more episodes of optic neuritis, and only one premature delivery was observed with no spontaneous abortion, neonatal malformations, or preeclampsia reported [9]. Given the limited data available in this setting, additional case reports remain valuable to better characterize the presentation and management of MOGAD in pregnant patients.

We report the case of a pregnant woman with acute visual loss and bilateral optic disc edema who was ultimately diagnosed with MOG-ON, highlighting the diagnostic challenges and clinical considerations of this condition during pregnancy.

## Case presentation

A 39-year-old woman, gravida 1 at 31 weeks of gestation, with a history of gestational diabetes and class III obesity (BMI 45.2), presented with a bifrontal headache described as stabbing and throbbing, initially rated 5/10 in intensity. On hospital admission, vital signs were as follows: blood pressure 130/80 mmHg, heart rate 72 beats per minute, respiratory rate 16 breaths per minute, and axillary temperature 36.7°C. Over the following three days, the headache progressively worsened, eventually reaching 10/10 intensity and becoming poorly responsive to analgesics.

Approximately three days after symptom onset, she noticed bilateral blurred vision accompanied by difficulty perceiving colors. These symptoms resolved spontaneously after about two days. Shortly thereafter, however, she developed sudden visual loss in the right eye, reporting that she could only perceive shadows. Due to the progression of visual symptoms, the patient was referred for neurological and ophthalmological evaluation.

Ophthalmological examination revealed bilateral papilledema. Visual assessment showed perception of shadows in the right eye with a relative afferent pupillary defect, while the left eye had a visual acuity of 20/100. On neurological examination, the patient was awake, oriented, and without motor or sensory deficits, although she reported pain with horizontal eye movements and tenderness with eyelid pressure.

**Figure 1:**
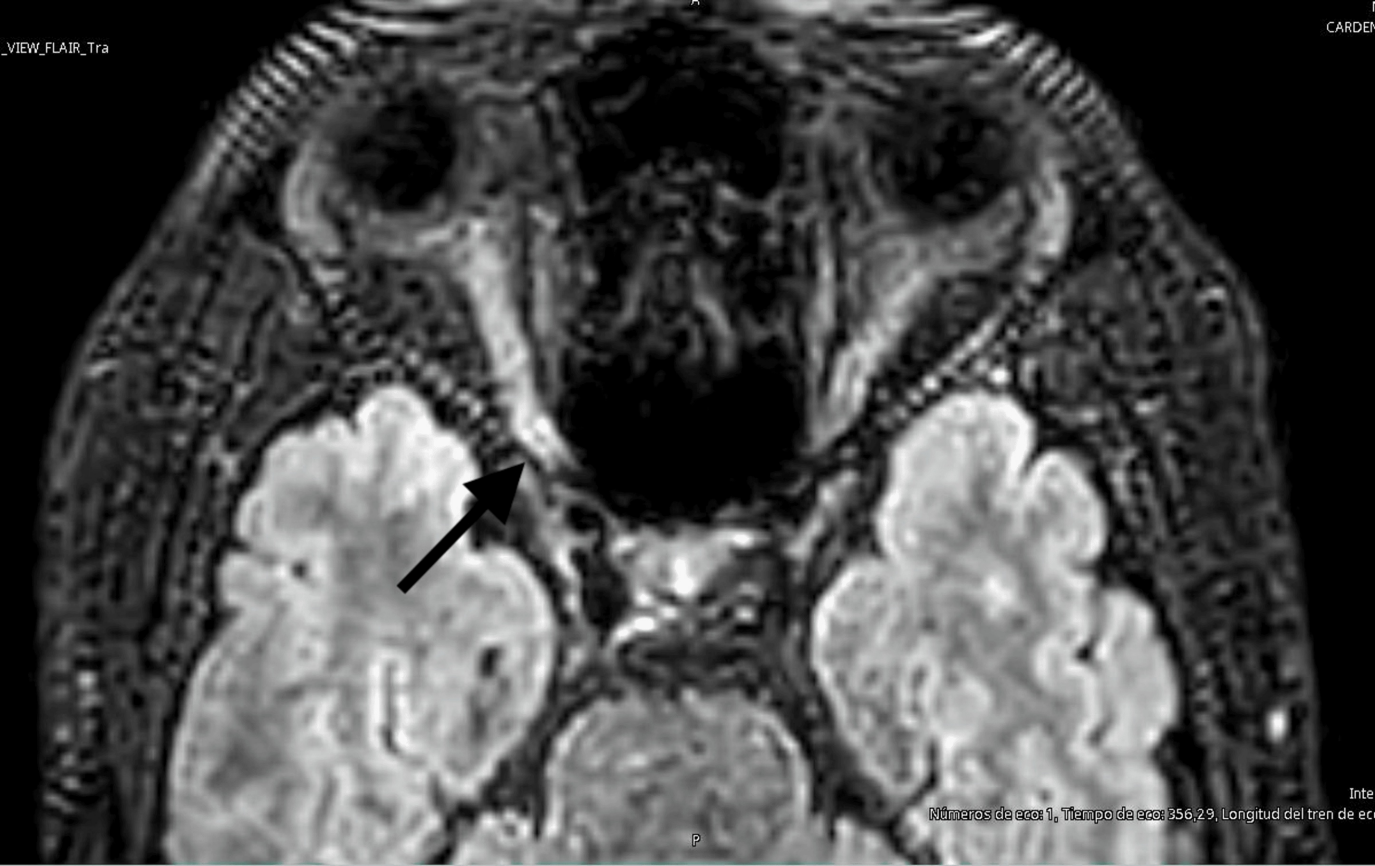
Brain MRI, non-contrast, T2-weighted FLAIR sequence, demonstrating hyperintensity in the intraorbital portion of the right optic nerve MRI: magnetic resonance imaging; FLAIR: fluid-attenuated inversion recovery

**Figure 2:**
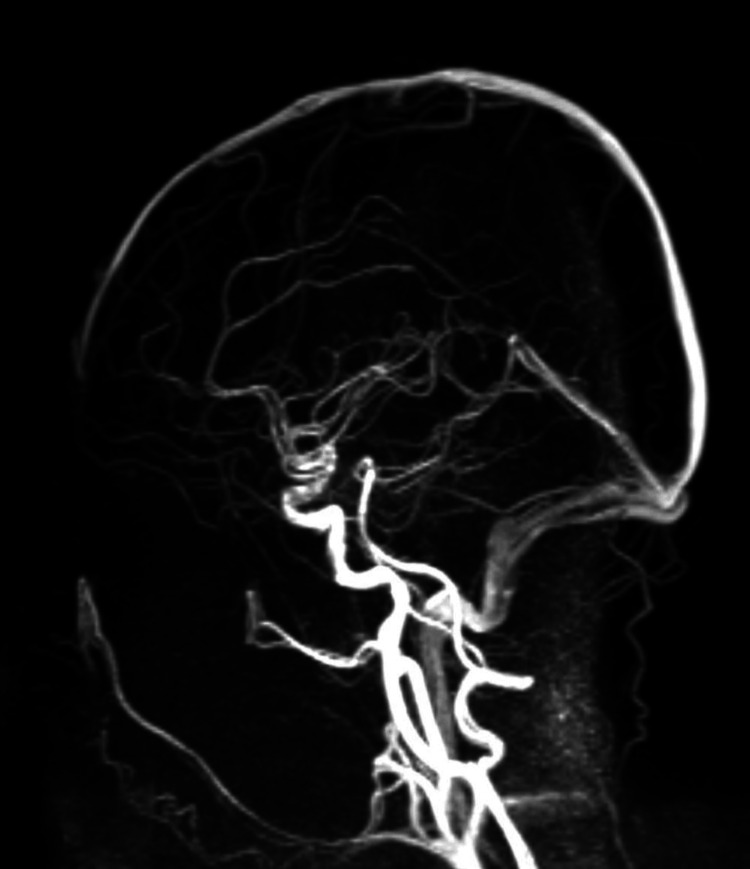
3D-TOF MR venography, sagittal reconstruction, demonstrating no evidence of cerebral venous thrombosis 3D-TOF: three-dimensional time-of-flight; MR venography: magnetic resonance venography

**Figure 3:**
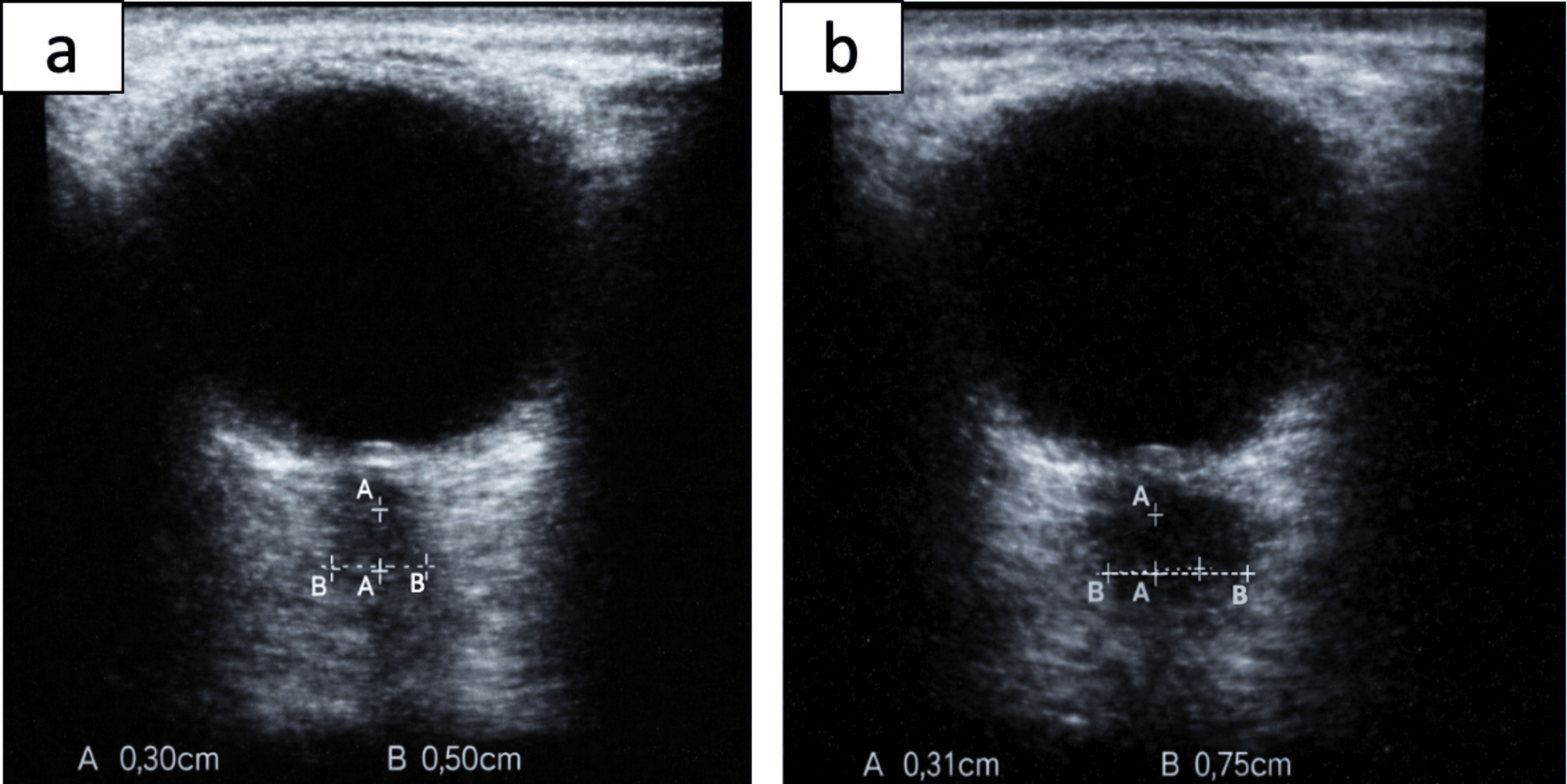
Ultrasound optic nerve sheath diameter measurement (diameter represented as B caliper length): (a) right eye and (b) left eye

At the brain level, MRI ruled out alterations in the hemispheres as well as in posterior fossa structures. In the intraorbital region, hyperintensity was detected along the right optic nerve in the fluid-attenuated inversion recovery (FLAIR) sequence (Figure 1), with no diffusion restriction or evidence of venous thrombosis (Figure 2). MRI of the spine showed no significant abnormalities. It was decided not to use contrast in this study considering the pregnancy, in addition to having other paraclinical tests that supported the diagnosis of optic neuritis. Ultrasound evaluation of the optic nerve sheath diameter revealed bilateral abnormalities; the normal diameter has an upper limit of 4.5 mm. In our patient, we found an optic nerve sheath diameter of 5 mm in the right eye and 7.5 mm in the left eye (Figure 3).

**Figure 4:**
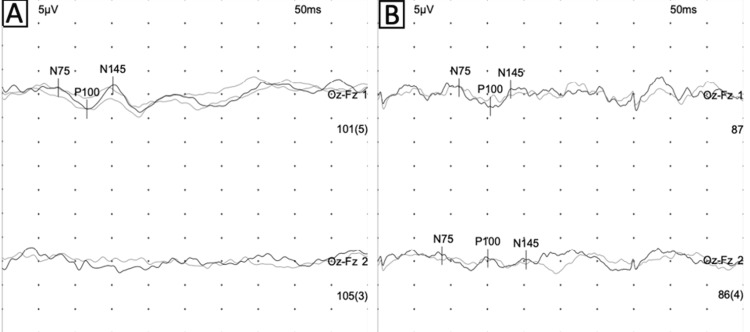
Visual evoked potentials: reverse pattern (A) and under stimulation with flash goggles technique (B) Numerical values on the right-inferior aspect of each graph represent average latencies for the P100 component and the number of averaged responses (inside parentheses). μV: microvolts; ms: milliseconds; Oz-Fz: midline occipital and frontal electrodes (Oz-Fz 1: left eye; Oz-Fz 2: right eye; N75/P100/N145: primary cortical visual sequence)

Visual evoked potential demonstrated prolonged latencies with reduced amplitudes, and when the right eye was evaluated, the reverse pattern P100 wave could not be accurately established, and it is clear that the waves have a significant decrease in amplitudes. These findings support the presence of an inflammatory process in the optic nerve (Figure 4).

A lumbar puncture (cerebrospinal fluid (CSF) sample is obtained by puncture with a Spinocan 22-French needle at the L3/L4 space level, performing an opening pressure measurement with a spinal manometer) was performed, ruling out the elevation of opening pressure with a reading of 19.5 cmH2O (the criterion for intracranial hypertension is greater than 22 cmH2O). The normal opening pressure of the CSF necessitates further diagnostic evaluation. CSF cytochemical parameters were normal, disregarding inflammatory processes associated with bacterial, fungal, or granulomatous infections. Oligoclonal bands were detected, with more than five bands present in CSF but not in a simultaneous serum sample (Table 1).

**Table 1:** Cytochemical analysis of CSF and oligoclonal bands *Positive result due to the presence of more than five gamma restriction bands that are not detected in a simultaneous serum sample. CSF: cerebrospinal fluid

CSF cytochemistry		Reference range
Aspect	Clear	-
Cellular count	0 leu/mm³	0-5 leu/mm³
India ink	Negative	-
Erythrocytes	Non-crenated erythrocyte	-
Gram stain	Negative	-
Glucose	57 mg/dL	45-80 mg/dL
Proteins	18 mg/dL	15-45 mg/dL
Chloride	125.3 mmol/L	118.1-132.0 mmol/L
Ph	8	-
Oligoclonal bands	Present*	-

Considering the clinical presentation, testing for demyelinating disorders was performed. Serum anti-MOG antibodies were positive, while AQP4, antinuclear antibody (ANA), anti-Ro/La, and anti-Smith antibodies were negative (Table 2). In this case, no lesions compatible with multiple sclerosis were detected, negative anti-aquaporin antibodies ruled out neuromyelitis optica, and with the presence of positive anti-MOG antibodies, the positivity of oligoclonal bands in CSF and negative in serum is considered an acute inflammatory component of MOGAD.

**Table 2:** Antibody titers and serum complement levels Normal levels: C3 80-180 mg/dL and C4 10-45 mg/dL. MOG: myelin oligodendrocyte glycoprotein; AQP4: aquaporin-4 channel: ANA: antinuclear antibody

Antibodies	Titer
Serum anti-MOG	1:10
Serum anti-AQP4	Negative
CSF anti-MOG	Negative
CSF anti-AQP4	Negative
ANAs	Negative
Anti-Ro	Negative
Anti-La	Negative
Anti-Smith	Negative
C3	178 mg/dL
C4	24 mg/dL

Given the evidence of the safety of intravenous steroid use in the third trimester of pregnancy, it was decided to initiate with intravenous methylprednisolone (500 mg daily for five days). No significant visual improvement was initially observed, with no light perception in the right eye and perception of hand movements in the left eye.

Four days after receiving the treatment, the patient began to show gradual visual improvement, recovering light perception in the right eye and the ability to count fingers at approximately three meters in the left eye. Given the partial response to corticosteroids, therapeutic plasma exchange was initiated (daily exchange for five days) and completed without maternal or fetal complications. Further ophthalmological evaluations demonstrated continued visual recovery, with reactive pupils but persistent bilateral papilledema. Visual acuity improved 2-3 days after the first plasma exchange session to 20/200 in the right eye and 20/50 in the left eye. It subsequently improved to 20/100 in the right eye and remained at 20/50 in the left eye, and near discharge reached 20/70 in the right eye and 20/30 in the left eye.

At the time of discharge, the patient showed clear improvement compared with admission, with near-complete recovery of vision in the left eye and partial recovery in the right eye. She was discharged on oral prednisone, with neurological follow-up and obstetric monitoring.

## Discussion

Studies of Reindl and Waters and Marignier et al. showed that MOGAD is an inflammatory demyelinating disorder of the central nervous system characterized by the presence of IgG antibodies directed against MOG [1,2]. Jurynczyk et al. demonstrated that, in adults, optic neuritis represents one of the most common clinical presentations [3], with the same conclusions in a Cobo-Calvo et al. revision in 2018 [4]. The diagnosis is established when a compatible clinical syndrome is accompanied by the detection of serum MOG-IgG using cell-based assays, published in international recommendations [5].

MOG-ON typically shows a recognizable clinical phenotype. As described by Chen and Bhatti, it is frequently bilateral and often accompanied by marked optic disc edema [6]. Orbital MRI commonly demonstrates longitudinally extensive involvement of the pre-chiasmatic optic nerve, frequently associated with the enhancement of the optic nerve sheath consistent with optic perineuritis. Clinically, patients often show a favorable initial response to corticosteroid therapy. However, Ramanathan et al. have reported that the disease course may be characterized by relapses, particularly when corticosteroids are rapidly tapered or discontinued [7].

Although orbital MRI often shows characteristic findings in MOG-ON, Chen and Bhatti note that imaging abnormalities may occasionally be subtle or absent, particularly early in the disease course, and therefore do not exclude the diagnosis [6]. As emphasized by Jarius et al., the diagnosis should be based on the clinical findings and supported by the serological detection of MOG-IgG antibodies; serological testing for MOG antibodies may be essential for establishing the correct diagnosis and guiding management [5]. In the case of our patient, we did not detect complete findings of optic nerve edema on MRI; further studies were conducted to confirm optic neuritis and subsequently to determine its etiology.

In the context of pregnancy, an important diagnostic challenge is that the bilateral optic disc edema observed in MOG-ON may mimic papilledema secondary to IIH. As highlighted by Chen and Bhatti, although both entities may share similar fundoscopic findings, MOG-ON typically presents with rapidly progressive visual loss accompanied by pain with eye movements, suggesting an inflammatory optic neuropathy [6]. At the same time, Marignier et al. have reported that MOGAD has occasionally been associated with elevated intracranial pressure and papilledema, further complicating the differential diagnosis [2].

Compared with optic neuritis associated with aquaporin-4 antibodies (AQP4-ON), Marignier et al. describe that visual recovery in MOG-ON is generally more favorable, particularly when corticosteroid treatment is initiated promptly [2]. Nevertheless, Ramanathan et al. emphasize that this initial responsiveness should not obscure the potential long-term impact of the disease, as recurrent inflammatory attacks may lead to cumulative optic nerve damage, including retinal nerve fiber layer loss, optic atrophy, and permanent visual impairment [7].

High-dose intravenous methylprednisolone remains the first-line therapy for acute attacks of MOGAD, including optic neuritis, as reported by Marignier et al. and Chen and Bhatti [2,6]. In cases of severe attacks or insufficient response to corticosteroids, plasma exchange or intravenous immunoglobulin (IVIG) may be considered as escalation therapies, as described by Chen and Bhatti [6]. Furthermore, Ramanathan et al. also recommend a prolonged oral corticosteroid taper, as rapid discontinuation has been associated with an increased risk of relapse [7].

Management of MOGAD during pregnancy and the postpartum period requires an individualized and multidisciplinary approach involving neurology, neuro-ophthalmology, and maternal-fetal medicine, largely due to the limited availability of disease-specific clinical trials in this population, as discussed by Marignier et al. [2]. For relapse prevention in the postpartum period, initiation or resumption of immunotherapy may be necessary. According to Marignier et al., available evidence regarding breastfeeding suggests a relatively reassuring safety profile for IVIG and azathioprine, while rituximab is increasingly considered compatible with breastfeeding according to expert recommendations, although treatment decisions should be individualized [2]. 

A systematic review by Cortese et al. suggests that pregnancy may exert a relatively protective effect on MOGAD activity. However, the postpartum period may be associated with an increased risk of relapse, although current evidence remains limited [9]. Additional reports remain valuable to further characterize the clinical behavior of this disease during pregnancy.

A cohort study by Bai et al., examining pregnancy-related optic neuritis, included 54 women and identified six patients with MOG-ON. In this series, only 16.4% of attacks occurred during pregnancy, whereas 83.6% developed within the first year postpartum or following abortion, with nearly half occurring during the first postpartum trimester. Notably, visual outcomes in MOG-ON were significantly better than those observed in patients with AQP4-ON [8].

The effects of MOGAD on reproduction are varied and can be considered across the preconception, pregnancy, and postpartum periods. As discussed by Cortese et al., fertility may be affected by residual symptoms related to spinal cord involvement or by prior immunosuppressive treatment. During pregnancy, adverse effects may occur secondary to residual sphincter dysfunction, leading to recurrent infections. Precise information regarding postpartum effects remains limited [9].

Diagnosis of MOGAD is established according to the criteria proposed by Banwell et al., the International MOGAD Panel [10], which require clinical evidence of a central demyelinating event, that is, optic neuritis, myelitis, acute disseminated encephalomyelitis (ADEM), monofocal or polyfocal deficits, brainstem or cerebellar deficits, or cortical encephalitis with or without seizures, together with a positive anti-MOG test and supportive radiological findings.

Treatment options for MOGAD and neuromyelitis optica spectrum disorders (NMOSD) include methylprednisolone, eculizumab, a monoclonal antibody targeting the C5 complement fragment approved for NMOSD, azathioprine, frequently used for MOGAD and NMOSD, and rituximab, a chimeric monoclonal antibody targeting the CD20 antigen on B lymphocytes, as described by Leite et al. [11].

A case report by Karalius et al. describes a 20-year-old woman at 31 weeks of gestation with a history of eye pain accompanied by decreased visual acuity in the right eye and subsequently in the left eye, along with color vision deficiencies. She was treated with 1 g methylprednisolone daily for five days. Serum AQP4 antibodies were tested, with negative results, while anti-MOG antibodies were positive at a 1:100 concentration. She was discharged with marked improvement after a gradual taper of oral steroids over two weeks. This is consistent with previous descriptions suggesting that steroid therapy is a safe option for MOGAD during pregnancy [12]. 

Finally, Tomizawa et al. have evaluated the role of positive oligoclonal bands in CSF in patients with MOGAD, which has been associated with a higher recurrence rate [13]. We will closely monitor our patient to establish early treatment during the postpartum period according to clinical evolution.

## Conclusions

MOG-ON should be considered in the differential diagnosis of acute visual loss during pregnancy. Although disease activity may decrease during gestation, the first clinical presentation can still occur during this period, and the risk of relapse appears to increase during the postpartum stage. Recognition of the characteristic clinical features, together with serological confirmation through MOG-IgG antibodies, is essential for establishing an accurate diagnosis and guiding appropriate clinical management. This case highlights the importance of maintaining a high index of suspicion when facing atypical presentations of optic disc edema during pregnancy and underscores the need for a comprehensive diagnostic and therapeutic approach. In our case, the presence of oligoclonal bands in CSF supports the need for periodic postpartum follow-up because of the higher risk of recurrence.

## References

[1] Reindl M, Waters P (2019). Myelin oligodendrocyte glycoprotein antibodies in neurological disease. Nat Rev Neurol.

[2] Marignier R, Hacohen Y, Cobo-Calvo Á (2021). Myelin-oligodendrocyte glycoprotein antibody-associated disease. Lancet Neurol.

[3] Jurynczyk M, Messina S, Woodhall MR (2017). Clinical presentation and prognosis in MOG-antibody disease: a UK study. Brain.

[4] Cobo-Calvo A, Ruiz A, Maillart E (2018). Clinical spectrum and prognostic value of CNS MOG autoimmunity in adults: the MOGADOR study. Neurology.

[5] Jarius S, Paul F, Aktas O (2018). MOG encephalomyelitis: international recommendations on diagnosis and antibody testing. J Neuroinflammation.

[6] Chen JJ, Bhatti MT (2020). Clinical phenotype, radiological features, and treatment of myelin oligodendrocyte glycoprotein-immunoglobulin G (MOG-IgG) optic neuritis. Curr Opin Neurol.

[7] Ramanathan S, Mohammad S, Tantsis E (2018). Clinical course, therapeutic responses and outcomes in relapsing MOG antibody-associated demyelination. J Neurol Neurosurg Psychiatry.

[8] Bai W, Sun M, Song H (2022). Serial analyses of clinical spectra and outcomes in Chinese women with pregnancy-induced optic neuritis. Front Med (Lausanne).

[9] Cortese R, Mariotto S, Mancinelli CR, Tortorella C (2022). Pregnancy and antibody-mediated CNS disorders: what do we know and what should we know?. Front Neurol.

[10] Banwell B, Bennett JL, Marignier R (2023). Diagnosis of myelin oligodendrocyte glycoprotein antibody-associated disease: international MOGAD panel proposed criteria. Lancet Neurol.

[11] Leite MI, Panahloo Z, Harrison N, Palace J (2023). A systematic literature review to examine the considerations around pregnancy in women of child-bearing age with myelin oligodendrocyte glycoprotein antibody-associated disease (MOGAD) or aquaporin 4 neuromyelitis optica spectrum disorder (AQP4+ NMOSD). Mult Scler Relat Disord.

[12] Karalius M, Mohan S, Paredes D, Rasool N, Bove R (2024). Pearls & oy-sters: optic neuritis as first demyelinating event during pregnancy in 2 young Hispanic women: MS vs MOGAD. Neurology.

[13] Tomizawa Y, Hoshino Y, Kamo R, Cossu D, Yokoyama K, Hattori N (2023). Comparing clinical and imaging features of patients with MOG antibody-positivity and with and without oligoclonal bands. Front Immunol.

